# Whole-Room Indirect Calorimetry in the Study of Obesity, Insulin Resistance and Metabolic Flexibility: A Narrative Review

**DOI:** 10.3390/nu18193287

**Published:** 2026-10-07

**Authors:** Andrii Zahorodnii, Alicja Jelska, Paulina Głuszyńska, Layth Majeed Mhayyal Almltashe, Hady Razak Hady

**Affiliations:** 1 Second Department of General and Gastroenterological Surgery, Medical University of Bialystok, 15-089 Bialystok, Poland; 2 Doctoral School, Medical University of Bialystok, 15-089 Bialystok, Poland

**Keywords:** whole-room indirect calorimetry, energy expenditure, respiratory exchange ratio, substrate oxidation, metabolic flexibility, obesity, insulin resistance, weight loss, bariatric surgery, anti-obesity pharmacotherapy

## Abstract

Obesity and insulin resistance are characterized by substantial heterogeneity in energy expenditure and substrate utilization. Whole-room indirect calorimetry (WRIC) enables continuous assessment of oxygen consumption, carbon dioxide production, energy expenditure, respiratory exchange ratio (RER), and substrate oxidation under controlled conditions. This narrative review examines the physiological interpretation, methodological strengths, limitations, and applications of WRIC in obesity, insulin resistance, metabolic flexibility, and weight-loss interventions. By enabling prolonged observation during resting, postprandial, exercise, and sleep periods, WRIC complements short-term indirect calorimetry and free-living energy expenditure measurements obtained using doubly labeled water. Validation studies generally demonstrate high technical accuracy and reproducibility for respiratory gas exchange and 24 h energy expenditure measured using WRIC. In contrast, estimates of the thermic effect of food, short-term changes in RER, and substrate oxidation are more sensitive to protocol design, energy balance, physical activity, and analytical procedures. Available studies demonstrate substantial interindividual variability rather than a uniform metabolic pattern associated with obesity or insulin resistance. Direct WRIC evidence concerning contemporary anti-obesity pharmacotherapy and metabolic and bariatric surgery remains limited. Standardized protocols and complementary assessments of body composition, insulin sensitivity, and free-living energy expenditure are needed to improve the comparability and interpretation of findings across studies.

## 1. Introduction

Obesity is a chronic and heterogeneous disease whose consequences extend beyond excessive adipose tissue accumulation [1,2,3]. It is frequently accompanied by adipose tissue dysfunction, chronic inflammation, dysregulated lipid metabolism, and progressive impairment of insulin sensitivity [4,5]. However, the severity of these abnormalities is not determined solely by body mass index (BMI) [2,3,6,7]. Fat distribution, including the visceral and subcutaneous adipose tissue ratio and the extent of ectopic lipid deposition, as well as fat-free mass and the individual capacity to respond to changes in energy supply and demand, also play important roles [6,7,8,9]. Consequently, individuals with a similar body weight may differ substantially in insulin sensitivity, energy expenditure and patterns of energy substrate utilization [6,7,10]. Therefore, body weight, BMI and routine laboratory parameters alone do not provide a comprehensive characterization of the metabolic phenotype [1,3,6,7].

Long-term body weight stability depends on the balance between energy intake and energy expenditure. Total energy expenditure (TEE) comprises resting energy expenditure (REE), the thermic effect of food (TEF), and physical activity energy expenditure (PAEE) [11,12,13]. However, metabolic assessment should consider not only the amount of energy expended but also the ability to adjust the oxidation of individual substrates to their availability and current energy demands. Between meals, when insulin concentrations are lower, the contribution of fat oxidation increases, whereas following carbohydrate-containing meals, the body should efficiently increase carbohydrate utilization. The ability to switch between predominant energy sources is referred to as metabolic flexibility (MF) [11,12,13]. In individuals with obesity, particularly those with coexisting insulin resistance (IR) and/or type 2 diabetes, this response may be attenuated or delayed, although its characteristics depend on the metabolic phenotype and the type of metabolic challenge applied [13,14,15].

Assessment of these changes requires methods capable of characterizing not only a single value of energy expenditure (EE), but also its temporal dynamics and the accompanying shifts in substrate utilization. Indirect calorimetry, based on the measurement of oxygen consumption (VO_2_) and carbon dioxide production (VCO_2_), enables the determination of EE and the respiratory exchange ratio (RER), as well as the estimation of carbohydrate and fat oxidation [16,17,18]. Protein oxidation may additionally be estimated when respiratory gas measurements are combined with an assessment of urinary nitrogen excretion [18,19]. Conventional short-term measurements using a face mask or ventilated-hood system primarily provide information on REE or the response to an acute intervention [16,20]. In contrast, whole-room indirect calorimetry (WRIC) permits continuous monitoring under controlled conditions while incorporating meals, scheduled physical activity, sedentary periods, and sleep into the study protocol. Depending on the research objective, the measurement period may range from several hours to 24 h or longer, enabling assessment of the magnitude and temporal pattern of the metabolic response [19,21].

Despite technological advances and the growing number of research centers using WRIC, comparisons across studies remain challenging. Protocols differ in measurement duration, procedures used to establish energy balance, meal composition and timing, prescribed physical activity, sleep conditions, and methods of data analysis and normalization [19]. These issues are particularly relevant in studies of weight-loss interventions because observed changes in EE may result from both the loss of metabolically active tissue and additional adaptive responses [16,22]. This review aims to summarize current knowledge on the application of WRIC in studies of obesity, IR, and MF. Particular attention is given to the interpretation of measured parameters, heterogeneity among study protocols, and the potential of WRIC to assess the metabolic consequences of weight loss. Evidence from dietary and lifestyle interventions, anti-obesity pharmacotherapy, metabolic and bariatric surgery, and combined treatment approaches will be evaluated where available.

### Literature Search Strategy

A structured literature search was conducted in PubMed/MEDLINE and Scopus. The original search was completed on 31 July 2026 and updated on 1 September 2026. The search covered publications from 1 January 2020 to the date of the final update. Terms describing whole-room calorimetry (“whole-room indirect calorimetry”, “whole-room calorimetry”, “whole-body room calorimeter”, “metabolic chamber”, and “room calorimeter”) were combined using Boolean operators with terms related to obesity, overweight, insulin resistance, insulin sensitivity, type 2 diabetes, metabolic flexibility, energy expenditure, respiratory exchange ratio, substrate oxidation, substrate balance, food quotient, controlled feeding, fasting, overfeeding, energy restriction, exercise, pharmacological weight loss, and bariatric surgery. A separate methodological search combined the calorimetry terms with “validation”, “calibration”, “accuracy”, “reliability”, “reproducibility”, “temporal response”, “standardization”, “methodology”, and “RICORS”. Eligible publications comprised English-language full-text articles involving adults aged ≥18 years. Both healthy individuals and participants with overweight, obesity, insulin resistance, type 2 diabetes, or other relevant metabolic phenotypes were considered. Original studies were eligible when they used whole-room indirect calorimetry (WRIC) to assess energy expenditure, respiratory gas exchange, respiratory exchange ratio, substrate oxidation, metabolic flexibility, or metabolic responses to nutritional, exercise, pharmacological, or surgical interventions. Methodological studies, reporting standards, systematic and scoping reviews, and other relevant reviews were used to support the technical and physiological context of the review. Direct WRIC evidence was defined as evidence obtained from studies in which participants remained inside a whole-room or whole-body open-circuit calorimeter and respiratory gas exchange was used to calculate chamber energy expenditure and/or substrate utilization over a defined measurement period [19]. Studies using ventilated-hood or ventilated-canopy indirect calorimetry, metabolic carts, or doubly labeled water were not classified as direct WRIC evidence. Such studies were considered only when they provided relevant complementary information and were explicitly identified as complementary evidence in the manuscript. Animal studies, studies involving exclusively participants younger than 18 years, conference abstracts without a corresponding full-text publication, non-English-language publications, and studies without outcomes relevant to the scope of the review were excluded. Studies published before 2020 were identified through backward screening of the reference lists of key WRIC publications and reviews, forward citation tracking, and targeted searches. Older publications were retained when they established an important methodological principle, described a validated measurement approach, or provided direct WRIC evidence not adequately represented in literature published from 2020 onwards.

Titles, abstracts, and full texts were initially assessed by A.Z. During manuscript revision, A.J. verified the eligibility of the included studies and their classification as direct WRIC or complementary evidence. Any uncertainties were resolved through discussion. Because of the narrative scope of the review and the substantial heterogeneity in study populations, chamber protocols, metabolic challenges, and analytical definitions, no formal risk-of-bias assessment, meta-analysis, or quantitative evidence synthesis was performed.

## 2. Whole-Room Indirect Calorimetry: Principles and Research Relevance

**Figure 1:**
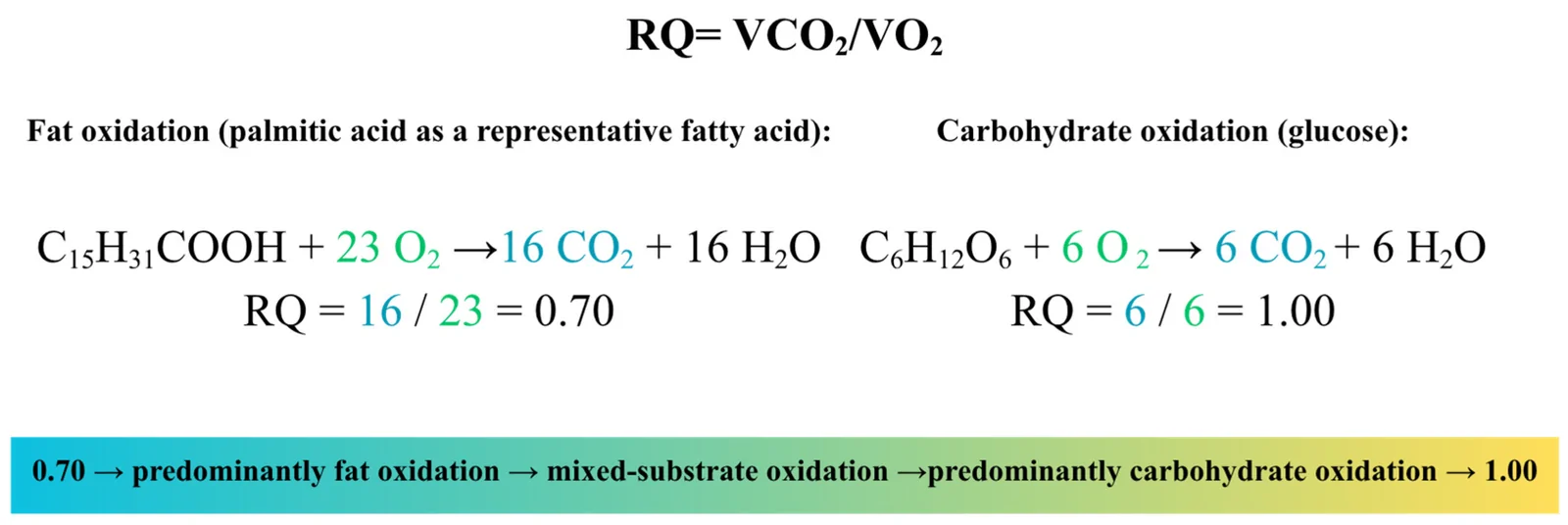
Stoichiometric basis of substrate-specific respiratory quotient. The values shown represent the theoretical RQ of fat and carbohydrate oxidation. Under metabolic steady-state conditions, whole-body RER approximates RQ.

An open-circuit whole-room calorimeter is a controlled, ventilated room in which oxygen consumption and carbon dioxide production are determined from the airflow and the differences in gas concentrations between incoming and outgoing air. These measurements are then used to calculate energy expenditure and the respiratory exchange ratio using indirect calorimetry equations, without requiring a respiratory mask or mouthpiece [17,19]. EE is typically calculated from measured VO_2_ and VCO_2_ using the modified Weir equation [16,17]. The ratio of VCO_2_ to VO_2_ measured at the whole-body level is referred to as the RER. The theoretical values associated with the oxidation of individual substrates derive from their respiratory quotient (RQ), defined as the ratio of CO_2_ produced to O_2_ consumed during substrate oxidation. The RQ is approximately 0.70 for fat and 1.00 for carbohydrate oxidation. Under metabolic steady-state conditions, whole-body RER approximates RQ and can therefore be used to estimate the predominant substrate being oxidized. However, RER and RQ may diverge during rapidly changing metabolic conditions, intense exercise, hyperventilation, or disturbances in acid–base balance [16,18]. Because protein oxidation cannot be reliably quantified from respiratory gas exchange alone, some protocols also measure urinary nitrogen excretion. Once protein oxidation has been accounted for, non-protein gas exchange data can be used to estimate carbohydrate and fat oxidation more accurately and to assess substrate balances relative to dietary intake. These estimates must nevertheless be interpreted in the context of overall energy balance, dietary composition, and the duration of the measurement period [18,19,23]. The stoichiometric basis and theoretical substrate-specific RQ values are illustrated in Figure 1.

The scope of information obtained using WRIC differs from that provided by other methods of assessing EE. Short-term indirect calorimetry using a face mask or ventilated hood provides high temporal resolution and is particularly useful for measuring REE and metabolic responses to acute interventions. However, these approaches may restrict movement and are not well suited to prolonged observation of metabolic changes after meals, during physical activity, or throughout sleep [16,20]. By contrast, the doubly labeled water (DLW) method enables the assessment of habitual free-living total daily EE over several days or weeks. However, it does not directly characterize individual components of energy expenditure, temporal metabolic responses, or substrate oxidation [16,24]. WRIC occupies an intermediate position, allowing detailed metabolic assessment over several hours or days under standardized conditions. Nevertheless, confinement to the calorimetry room may alter habitual patterns of physical activity, sleep, and eating behavior. These methods should therefore be regarded as complementary rather than interchangeable, and the choice of method should be guided by the primary research question [16,19,21].

## 3. Energy Metabolism in Obesity and Insulin Resistance

### 3.1. Components and Determinants of Energy Expenditure

REE generally represents the largest component of TEE, accounting for approximately 60–75% of TEE in individuals with a sedentary lifestyle. Its primary determinant is fat-free mass (FFM), although FFM comprises tissues with markedly different metabolic activities: internal organs expend substantially more energy per unit mass than skeletal muscle. REE is also influenced by fat mass, age, sex, hormonal status, ambient temperature, and current energy balance [16,25,26,27]. TEF typically accounts for approximately 10% of TEE, and its magnitude depends on the amount and composition of food consumed; protein elicits the greatest response, followed by carbohydrates, whereas fat produces the smallest response [25,28]. PAEE is the most variable component of TEE and depends on both the participant’s activity level and the conditions imposed by the study protocol. Consequently, the relative contributions of these components differ among individuals and may change during weight loss or other metabolic interventions [16,25,29].

### 3.2. Respiratory Exchange Ratio and Substrate Oxidation

Under appropriate metabolic conditions, a single RER value may provide an estimate of the predominant substrate being oxidized, whereas its interpretation over a longer period can be extended by comparison with the food quotient (FQ). The FQ represents the weighted theoretical RQ of the macronutrients contained in the consumed diet. Under conditions approximating energy balance and over a sufficiently long measurement period, a mean RER similar to the FQ suggests that the relative proportions of oxidized substrates broadly correspond to their dietary intake. A sustained mean RER above the FQ may indicate lower fat oxidation relative to dietary fat intake, whereas a mean RER below the FQ may indicate a greater relative contribution of fat oxidation. However, the RER–FQ difference alone does not establish a positive or negative fat balance because its interpretation also depends on overall energy balance, protein oxidation and balance, dietary composition, and measurement duration [19,23,30]. Together, temporal RER measurements and comparison with the FQ enable assessment of short-term substrate selection and the extent to which metabolism adapts to dietary composition over several hours or an entire day [31,32].

### 3.3. Metabolic Flexibility and Insulin Resistance

MF is a dynamic trait and should not be assessed from a single fasting RER value. It is best quantified as the magnitude and rate of change in RER or substrate oxidation in response to a standardized metabolic challenge [12,14,33]. The classical approach measures the change in RER during a hyperinsulinemic–euglycemic clamp, thereby assessing the ability to shift from fat oxidation toward increased glucose utilization in response to insulin [33,34]. Meal challenges examine this response under more physiological conditions, whereas exercise-based assessments evaluate substrate selection across changing energy demands. WRIC complements these approaches by enabling prolonged assessment of RER dynamics during standardized feeding, fasting, exercise, sleep, and changes in energy balance [31,32,35]. These whole-body responses reflect coordinated regulation across several tissues. During fasting, lower insulin concentrations promote adipose-tissue lipolysis and fatty-acid availability, while hepatic glucose production maintains circulating glucose. Following feeding or insulin stimulation, suppression of lipolysis and hepatic glucose production, together with increased glucose uptake and oxidation, shifts substrate utilization toward carbohydrates. The magnitude and rate of this transition may also be influenced by skeletal-muscle mitochondrial oxidative capacity and coupling. In insulin resistance, disruption of these coordinated responses may attenuate or delay substrate switching [11,13,33]. WRIC captures the integrated whole-body consequences of these processes but cannot identify the contribution of individual organs or tissues. Moreover, the apparent magnitude of metabolic inflexibility depends on the metabolic challenge, baseline metabolic phenotype, adiposity, insulin sensitivity, and analytical definition. Results derived from fasting, meal, clamp, exercise, and sleep protocols should therefore be considered complementary rather than directly interchangeable [12,14,31,34].

### 3.4. Body Composition, Energy Balance and Metabolic Adaptation

Reductions in REE and TEE during weight loss are largely expected consequences of tissue loss, particularly the loss of FFM, together with decreases in the energy cost of physical activity and TEF. When the observed reduction in EE exceeds the value predicted from the current body weight and composition, this difference is referred to as adaptive thermogenesis or metabolic adaptation [16,22,36]. This response may involve changes in sympathetic nervous system activity, thyroid function, leptin and insulin concentrations, and the efficiency of cellular processes [37,38,39,40]. However, its magnitude depends on the timing of measurement. An ongoing energy deficit may transiently reduce EE and increase fat oxidation independently of the weight loss already achieved. Therefore, measurements performed before and after an intervention should be conducted under comparable conditions of energy balance, dietary intake, and physical activity [19,22,36,41]. Combining WRIC with body-composition assessment helps determine which proportion of the change in EE results from tissue loss and which may represent an additional adaptive response [16,19,22]. The principal parameters measured or derived using WRIC and their physiological interpretation are summarized in Table 1.

## 4. Applications of WRIC in Obesity and Weight-Loss Research

The principal application studies using WRIC to investigate metabolic flexibility, nutritional challenges, exercise, pharmacological weight loss, and bariatric surgery are summarized in Supplementary Table S1. Characteristics of principal studies using whole-room indirect calorimetry to investigate metabolic flexibility and weight-loss interventions. These studies differ substantially in population characteristics, chamber protocols, metabolic challenges, outcome definitions, and analytical approaches, which should be considered when comparing their findings.

### 4.1. Resting, Sleeping and 24 h Energy Expenditure

Resting, sleeping, and 24 h EE measured using WRIC describe partly distinct aspects of human metabolism. REE refers to EE under defined resting conditions, whereas sleeping energy expenditure (SEE) minimizes the contributions of conscious activity and arousal-related responses. In contrast, 24 h EE measured using WRIC integrates the effects of rest, sleep, food intake, and physical activity performed during the measurement protocol. It should not be considered equivalent to habitual free-living total daily EE assessed using doubly labeled water, which incorporates spontaneous and planned activities performed outside the controlled WRIC environment [16,19,24]. Comparisons of EE between individuals with different metabolic phenotypes require adjustment for body size and composition. Fat-free mass is a major determinant of resting and 24 h EE, with an additional contribution from fat mass. Consequently, higher absolute EE should not itself be interpreted as lower metabolic efficiency, nor should lower absolute EE be taken as evidence of metabolic slowing. Regression-based adjustment is preferable to expressing EE per kilogram of body weight or fat-free mass when comparing groups with different body composition [19]. Seminal chamber studies established that body-composition-adjusted 24 h EE and 24 h RQ are associated with subsequent weight change [42,43]. In Pima Indians, lower adjusted 24 h EE and higher 24 h RQ predicted greater subsequent weight gain [42,43]. In a separate caloric-restriction study, the magnitude of the EE response to fasting and overfeeding predicted subsequent weight loss, supporting the concept of distinct “thrifty” and “spendthrift” metabolic phenotypes [44]. Longer-term data from the CALERIE trial further demonstrated sustained metabolic adaptation during approximately 15% caloric restriction over two years [45]. WRIC studies comparing different metabolic phenotypes suggest that differences may be more apparent in the temporal pattern of metabolic responses than in mean 24 h EE alone. Carnero et al. compared 24 h substrate-oxidation patterns among physically active lean individuals, individuals with obesity without diabetes, and individuals with obesity and type 2 diabetes. They observed differences in sleeping and postprandial substrate-oxidation responses, while aerobic training improved selected postprandial responses in participants with type 2 diabetes, although the individual indices of metabolic flexibility did not change uniformly [35]. These findings indicate that EE and the capacity to shift substrate utilization are related but distinct metabolic characteristics. The absence of a difference in mean 24 h EE does not exclude an altered sleeping or postprandial response, whereas an isolated REE measurement cannot characterize the temporal dynamics of whole-day metabolism. The principal application studies using WRIC and the differences between their protocols are summarized in Supplementary Table S1. Characteristics of principal studies using whole-room indirect calorimetry to investigate metabolic flexibility and weight-loss interventions.

### 4.2. Metabolic Flexibility and Controlled Nutritional Interventions

WRIC enables the assessment of changes in substrate utilization during fasting, overfeeding, and controlled interventions differing in energy content and macronutrient composition. Meal timing may also modify substrate utilization independently of total daily energy intake. In a controlled early time-restricted feeding study, 24 h WRIC showed no significant change in total energy expenditure, but earlier food intake increased fat oxidation and improved appetite-related outcomes [46]. Complementary inpatient chamber studies comparing diets differing in macronutrient composition demonstrated that dietary carbohydrate and fat content influence energy expenditure and substrate oxidation, although these findings should be distinguished from effects observed under free-living conditions [47,48]. Studies by Begaye et al. and Unlu et al. demonstrated substantial interindividual variability in RER responses to fasting and overfeeding. A smaller reduction in RER during high-fat overfeeding was associated with greater subsequent weight gain, whereas smaller fasting-induced reductions in RER and carbohydrate oxidation were associated with greater subsequent ad libitum energy intake [49,50]. However, these associations were observational and do not establish that impaired metabolic flexibility directly causes a positive energy balance. Perry et al. subsequently examined repeated 24 h nutritional interventions differing in fat, carbohydrate, and protein content. The study demonstrated the expected changes in RER and substrate oxidation according to macronutrient availability but also revealed considerable interindividual heterogeneity [51]. Begaye et al. and Perry et al. report analyses from the same registered study (NCT00523627), whereas Unlu et al. report a separately registered study (NCT02939404) [49,50,51]. The exact extent of participant overlap across these reports could not be established from the information reviewed; therefore, complete independence of the study samples should not be assumed. The composition of an individual meal also influences the temporal pattern of RER and substrate oxidation. McDougal et al. demonstrated different overnight responses following isocaloric high-fat and low-fat dinners [31]. In a subsequent study, selected indices of the response to a high-fat meal were reproducible but did not correlate with metabolic flexibility assessed using a hyperinsulinemic–euglycemic clamp [32]. These findings indicate that responses to fasting, dietary fat, and insulin-stimulated glucose utilization represent different aspects of metabolic flexibility and should not be interpreted as interchangeable measurements. Interpretation additionally requires consideration of energy balance, dietary composition, food quotient, preceding physical activity, and measurement duration [12,19]. RER values may exceed 1.00 during net de novo lipogenesis after marked carbohydrate overfeeding and may fall below 0.70 during ketosis or when gluconeogenesis contributes substantially to substrate metabolism; such values should therefore be interpreted in relation to the prevailing energy balance and metabolic state [18,19].

### 4.3. Dietary Energy Restriction and Exercise Interventions

**Figure 2:**
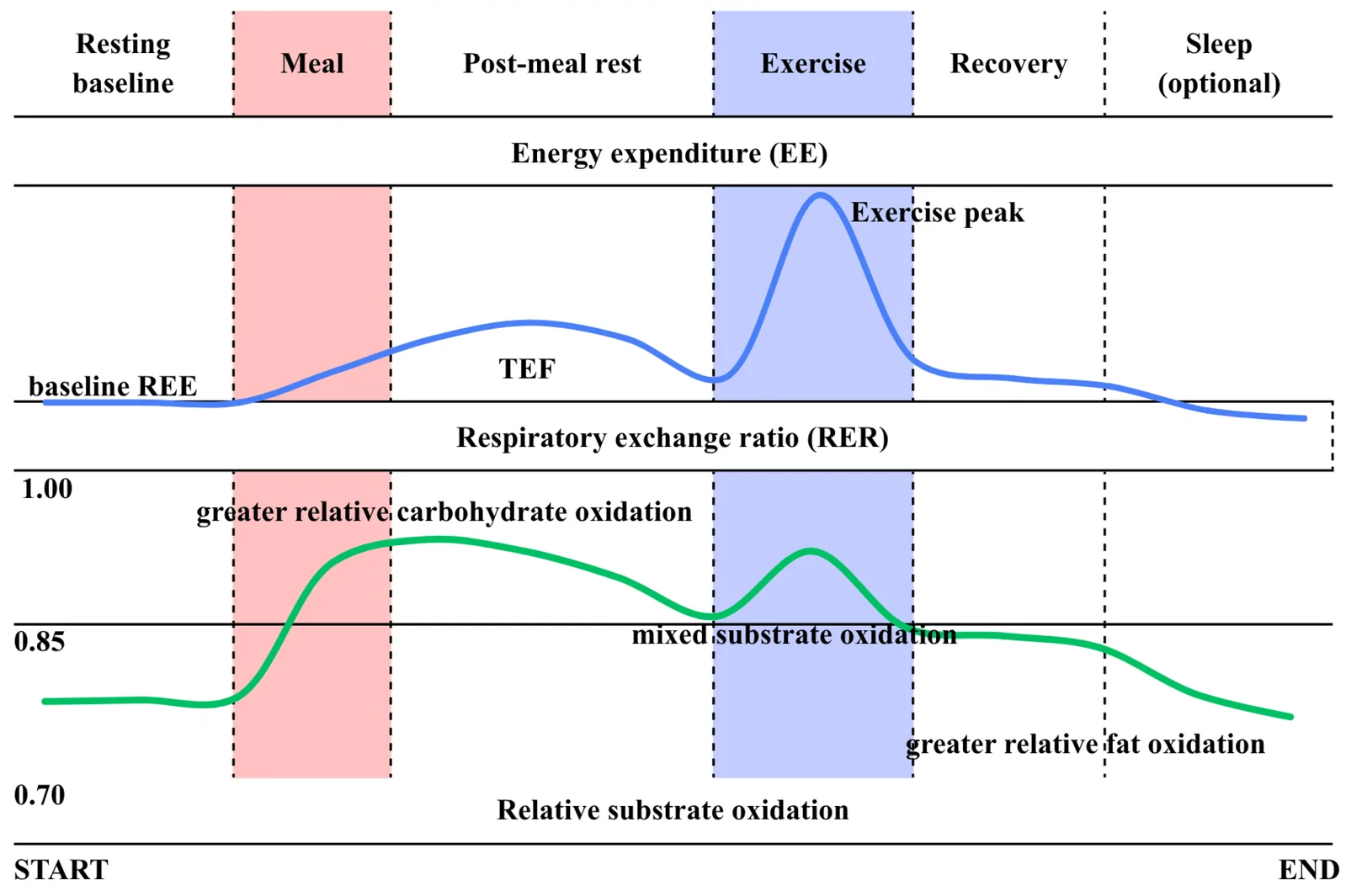
Illustrative temporal changes in energy expenditure, RER, and relative substrate oxidation during a standardized WRIC protocol. The direction and magnitude of substrate shifts depend on nutritional, exercise-related, and metabolic factors. The curves are schematic and do not represent empirical data. The shaded regions indicate the meal and exercise phases of the illustrative protocol.

WRIC can characterize changes in EE and substrate utilization during active energy restriction and after weight stabilization. These phases should be distinguished because a negative energy balance may temporarily reduce EE and increase fat oxidation independently of longer-term changes in body weight or composition [22,36,41]. In a small inpatient study, Heinitz et al. observed that adaptive thermogenesis in 24 h EE appeared within the first week of 50% caloric restriction and was associated with less subsequent weight loss [41]. However, the exploratory design and small sample size limit the generalizability of these findings. Comparisons before and after weight loss should therefore account for changes in fat-free and fat mass, the achieved energy balance, and the timing of the WRIC assessment [19,22]. Exercise interventions may affect EE during and after exercise, as well as the temporal pattern of substrate utilization. In adults with obesity and type 2 diabetes, aerobic training improved selected postprandial indices of metabolic flexibility, although not all WRIC-derived measures changed consistently [35]. In a randomized crossover study of nine healthy young men, Babu et al. found that post-dinner exercise increased overnight energy expenditure and fat oxidation compared with conditions without exercise [52]. Current evidence therefore does not support a single characteristic WRIC response to lifestyle-induced weight loss. Standardized longitudinal studies combining controlled energy intake, body-composition assessment, and repeated WRIC measurements during active weight loss and weight maintenance are needed to distinguish transient energy-deficit responses from persistent metabolic adaptations [19,22]. An illustrative temporal profile of EE, RER, and relative substrate oxidation during a standardized WRIC protocol is presented in Figure 2.

### 4.4. Pharmacological Interventions in Obesity

Direct WRIC evidence concerning the effects of anti-obesity pharmacotherapy on energy metabolism remains limited. A recent scoping review identified only four studies using 24 h WRIC among 23 studies examining the effects of GLP-1 receptor agonists or multi-receptor agonists on EE [53]. These chamber studies investigated exenatide, liraglutide, GLP-1/glucagon co-agonism, and tirzepatide. Overall, they did not demonstrate a consistent increase in 24 h chamber EE independent of changes in body weight and composition, although some reported greater fat oxidation or reduced metabolic adaptation under controlled conditions [54,55,56,57]. Interpretation is limited by small sample sizes and differences in treatment duration, energy restriction, achieved weight loss, energy balance during measurement, and adjustment for body composition. Studies assessing appetite, ad libitum energy intake, or body composition without calorimetry provide relevant complementary context but cannot establish an effect on EE [58]. Direct WRIC studies of semaglutide, head-to-head comparisons of different anti-obesity medications, and assessments of TEF remain lacking [53]. Future studies should include repeated WRIC measurements during active weight loss and after weight stabilization and should compare interventions at similar levels of energy deficit, weight loss, and body composition.

### 4.5. Metabolic and Bariatric Surgery

Evidence concerning energy metabolism after metabolic and bariatric surgery has been obtained using several distinct methods, including short-term indirect calorimetry, doubly labeled water, and, less frequently, WRIC. Measurements using a ventilated hood or canopy primarily characterize REE, whereas doubly labeled water estimates habitual free-living total daily EE without characterizing sleeping, postprandial, or substrate-specific responses. These methods provide complementary information but should not be interpreted as interchangeable [16,24]. In the principal direct WRIC study, Tam et al. compared changes in 24 h and sleeping EE following Roux-en-Y gastric bypass, sleeve gastrectomy, laparoscopic adjustable gastric banding, and diet-induced weight loss. After adjustment for changes in body composition, 24 h EE decreased in all groups after eight weeks, whereas the reduction persisted at one year after Roux-en-Y gastric bypass and sleeve gastrectomy. However, the magnitude of metabolic adaptation did not differ significantly between treatment groups [59]. Interpretation is limited by the small, non-randomized groups and differences in the degree of weight loss. Complementary studies using short-term indirect calorimetry have examined changes in REE after bariatric surgery, whereas doubly labeled water studies have provided information on free-living total daily EE [60,61,62,63]. Although relevant to the broader assessment of metabolic adaptation, these studies cannot establish procedure-specific differences in 24 h chamber EE, sleeping EE, TEF, or substrate oxidation. Further prospective WRIC studies using standardized protocols and comparable stages of weight loss are therefore required to determine whether individual surgical procedures or the addition of anti-obesity pharmacotherapy produce distinct metabolic responses.

## 5. Methodological Challenges and Future Research

### 5.1. Technical Accuracy and Sources of Measurement Error

The accuracy of WRIC depends on the performance and stability of the entire measurement system rather than on gas-analyzer accuracy alone. Reliable calculation of VO_2_ and VCO_2_ requires accurate measurement of airflow, adequate room airtightness, stable environmental conditions, and appropriate correction for temperature, humidity, and atmospheric pressure. Analyzer drift or small errors in airflow measurement may propagate directly into calculated VO_2_, VCO_2_, EE, and RER. Regular calibration and validation using controlled gas infusion or combustion of substances with known stoichiometry are therefore essential [19,64,65,66]. An important technical limitation is the temporal response of the system. Because expired gases are diluted within a relatively large chamber volume, changes in gas exchange are not detected instantaneously. System response depends on chamber volume, airflow rate, efficiency of air mixing, gas-sampling transit time, and analyzer response time. These factors may smooth or delay short-lived metabolic signals and are particularly relevant when small or rapidly changing responses, such as the TEF or brief exercise-induced changes, are evaluated [19,67,68]. Accurate estimation of gas exchange also requires representative sampling of chamber air. Incomplete mixing or spatial gradients in gas concentration may cause the sampled air to differ from the mean gas composition within the room. The ventilation layout, location of sampling points, effective chamber volume, and dynamic recovery of infused gases should therefore be evaluated during commissioning and periodic validation of the system [19,64,66]. Door opening, use of transfer ports, temporary equipment malfunction, or the introduction of uncontrolled sources of carbon dioxide may disturb the gas balance and generate missing or unreliable data. Such events should be minimized, time-stamped, and considered during data processing. Studies should report calibration procedures, the frequency of quality-control testing, system response time, temporal resolution, procedures for handling measurement disturbances, and the minimum detectable difference for the principal outcomes [19,67]. WRIC systems vary in room dimensions, effective volume, airflow configuration (push, pull, or push–pull), gas analyzers, environmental-control systems, and signal-processing methods. Although many systems are custom-built, commercial modular and turnkey solutions are also available. RICORS 1.0 does not specify a minimum or maximum room volume; instead, the effective volume should be accurately determined and reported because, together with airflow, it influences system response time. Published studies also differ in the reporting of calibration and temporal performance. RICORS therefore recommends calibration against traceable standards, regular zero and combustion tests, dynamic gas-infusion testing when short-term responses are evaluated, and reporting of measurement accuracy, precision, response time, and data-processing methods [19,21,68].

### 5.2. Protocol Standardization and Interpretation of Results

The comparability of findings depends not only on how the measurement is conducted but also on how individual outcomes are defined. Sleeping EE may be calculated as the mean value over the entire sleep period or from the lowest stable measurement interval. TEF is estimated as the cumulative increase in EE following a standardized meal relative to baseline REE, with physical activity either minimized or accounted for analytically. Outcomes reported under the same name may therefore not be directly comparable when different calculation methods are used [19,28,67]. Study protocols should specify the analyzed time intervals, methods of temporal averaging, criteria for excluding measurement artifacts, and procedures used to account for physical activity. The timing and composition of meals, fasting duration, sleep schedule, and environmental conditions should remain consistent across repeated measurements. Diet and physical activity during the days preceding the assessment should also be standardized, because differences in glycogen availability and recent energy intake may affect RER and substrate oxidation independently of the intervention being studied [19,30,31]. The actual energy balance achieved during the WRIC stay should be reported, as even an unintended energy deficit or surplus may alter substrate oxidation [23,30]. Adherence to standardized operating and reporting guidance, such as the Room Indirect Calorimetry Operating and Reporting Standards (RICORS 1.0), facilitates reliable comparisons across research centers [19]. RICORS 1.0 establishes core reporting standards but does not prescribe every environmental or participant-related characteristic. Temperature, humidity, lighting, age eligibility, and psychological criteria should therefore be controlled and reported according to the study objective and protocol [19]. Eligibility criteria should be study-specific and should additionally consider the participant’s ability to remain in an enclosed environment and comply with prolonged standardized feeding, activity, and sleep procedures [19]. WRIC protocols necessarily restrict habitual food intake and spontaneous physical activity and therefore should not be interpreted as direct representations of free-living behavior. Their principal value lies in the use of standardized conditions and metabolic challenges that enable characterization of individual patterns of energy expenditure, substrate utilization, and metabolic switching. These controlled responses may reveal metabolic tendencies but should be complemented by free-living measurements when habitual behavior or total daily energy expenditure is the outcome of interest [16,19,24].

### 5.3. Priorities for Future Research on Obesity and Weight Loss

Future studies should employ prospective longitudinal protocols with repeated WRIC measurements in the same participants before treatment initiation, during active weight loss, and after a period of weight stabilization. Such designs would help distinguish transient metabolic effects of an ongoing energy deficit from adaptations that persist after active weight loss has ended. Measurements at each time point should be conducted under comparable conditions of energy balance, dietary composition, physical activity, and sleep [19,22,36,41]. The duration of each WRIC stay and the number of repeated measurement days should be determined by the prespecified primary outcome. Highly reproducible outcomes such as 24 h EE and sleeping EE may require fewer repeated measurements than variables with greater within-person variability. In particular, TEF and short-term RER responses may require repeated assessments, larger samples, or analytical methods capable of accounting for temporal variability [19,65,66,67]. Validation studies using propane combustion and repeated human measurements indicate high technical accuracy and reproducibility of WRIC-derived gas exchange and 24 h EE. Reported within-subject coefficients of variation are generally approximately 1–5% for 24 h EE and higher for substrate oxidation, commonly ranging from 5% to 25%. In one repeated-measures study, reliability was 97.8% for 24 h EE and 96.8% for sleeping EE, whereas reliability was lower for some components such as substrate oxidation and the thermic effect of food. Technical validation studies have generally demonstrated close agreement between measured and expected VO_2_, VCO_2_, and EE values, but the precision of short-term RER responses and TEF estimates remains more sensitive to protocol duration, meal composition, and analytical procedures [64,65,66,67]. Interpretation of longitudinal changes would be strengthened by concurrent assessment of body composition, insulin sensitivity, circulating metabolic and appetite-regulating hormones, and actual energy intake. Combining WRIC with doubly labeled water may additionally clarify whether metabolic responses observed under standardized laboratory conditions translate into changes in average free-living total daily EE [16,19,24]. Further studies should directly compare modern anti-obesity pharmacotherapy, metabolic and bariatric surgery, and combined treatment strategies using standardized WRIC protocols and comparable degrees of weight loss. Particular priorities include head-to-head comparisons of different pharmacological agents and bariatric procedures, assessment of pharmacotherapy initiated after surgery, and longer follow-up extending beyond the active weight-loss phase. Current WRIC evidence for these interventions remains limited, particularly for contemporary incretin-based therapies and combined surgical–pharmacological treatment [53,57,59,69].

## 6. Conclusions

WRIC provides a detailed assessment of energy metabolism under controlled conditions by enabling continuous evaluation of respiratory gas exchange, 24 h chamber EE, RER, and substrate oxidation during resting, postprandial, activity, and sleep periods. Validation studies generally demonstrate high technical accuracy and reproducibility for respiratory gas exchange and 24 h chamber EE, whereas estimates of TEF, short-term changes in RER, and substrate oxidation are more sensitive to protocol design, energy balance, physical activity, and analytical methods. Available evidence supports the value of WRIC for metabolic phenotyping but does not indicate a single pattern of EE or metabolic flexibility shared by all individuals with obesity, insulin resistance, or type 2 diabetes. Direct WRIC evidence concerning contemporary anti-obesity pharmacotherapy and metabolic and bariatric surgery remains limited. Findings obtained using short-term indirect calorimetry or doubly labeled water provide complementary information but should not be interpreted as directly equivalent to WRIC measurements. Combining WRIC with body-composition assessment, measures of insulin sensitivity, and free-living total daily EE assessed using doubly labeled water may provide a more comprehensive characterization of metabolic responses. Further standardized, prospective, and multicenter studies are required to improve comparability and determine the role of WRIC in obesity and weight-loss research.

## Figures and Tables

**Table 1:** Main parameters measured or derived using WRIC and their physiological interpretation. Source: Authors’ compilation based on Chen et al. [19], Fernández-Verdejo et al. [16], Hall and Guo [23], Weir [17], and Frayn [18].

Parameter	Method of Determination	Physiological Significance	Key Limitation
VO_2_ (oxygen consumption)	Measurement of oxygen consumption	Reflects the oxygen demand of oxidative metabolic processes	Depends on the accuracy of airflow and gas-concentration measurements
VCO_2_ (carbon dioxide production)	Measurement of carbon dioxide production	Reflects the amount of CO_2_ generated and eliminated during metabolism	May be influenced by ventilation and acid–base balance
RER (respiratory exchange ratio)	Calculated as VCO_2_/VO_2_	Indicates the relative contribution of fat and carbohydrate oxidation	Most informative under metabolically stable conditions
EE (energy expenditure)	Calculated from VO_2_ and VCO_2_ (using the Weir equation)	Quantifies the amount of energy expended over a defined period	Accuracy depends on gas exchange measurements and the equation applied
REE (resting energy expenditure)	EE measured during standard resting period	Shows the energy cost of maintaining basic physiological functions at rest	Requires control of posture, low-level physical activity, ambient temperature
Sleeping EE (energy expenditure)	Averaged over selected metabolically stable periods during sleep	Enables assessment of metabolism during minimal activity	Depends on the study protocol and study duration
Carbohydrate oxidation	Stoichiometric calculation based on VO_2,_ VCO_2_ and urinary nitrogen excretion	Quantifies the rate of carbohydrate utilization as an energy source	Requires adjustment for protein oxidation
Fat oxidation	Stoichiometric calculation based on VO_2,_ VCO_2_ and urinary nitrogen excretion	Quantifies the rate of fat utilization as an energy source	Requires adjustment for protein oxidation
Energy and substrate balances	Difference between energy/substrate intake and expenditure or oxidation	Indicates the direction of energy storage or mobilization	Requires accurate assessment of intake and a long measurement period

## Data Availability

No new data were created or analyzed in this study. Data sharing is not applicable to this article.

## Supplementary Materials

The following supporting information can be downloaded at: https://www.mdpi.com/article/10.3390/nu18193287/s1. Supplementary Table S1. Characteristics of principal studies using whole-room indirect calorimetry to investigate metabolic flexibility and weight-loss interventions.
