# Supplementary material for: Whole-Room Indirect Calorimetry in the Study of Obesity, Insulin Resistance and Metabolic Flexibility: A Narrative Review
Source: Nutrients. 2026 Oct 7;18(19):3287. doi: 10.3390/nu18193287 (PMC13667147; doi:10.3390/nu18193287)
Supplement: Supplementary file 1 [file nutrients-18-03287-s001.zip › nutrients-4550503-supplementary.pdf]

Supplementary Table S1. Characteristics of principal studies using whole-room indirect calorimetry to investigate metabolic flexibility and weight-loss interventions.

Study	Population	Intervention or metabolic challenge	WRIC protocol	Main outcomes	Principal findings	Major limitation
Begaye et al., 2020 [49]	79 healthy, weight-stable adults with normal glucose regulation	Energy balance, 24-h fasting, and three 200% overfeeding diets differing in macronutrient composition	Repeated 24-h chamber assessments under five controlled dietary conditions in a crossover design	24-h EE, RQ, carbohydrate and fat oxidation, and free-living weight change at 6 and 12 months	RQ decreased during fasting and high-fat overfeeding but increased during standard and high-carbohydrate overfeeding. A smaller reduction in RQ and a smaller increase in fat oxidation during high-fat overfeeding predicted greater subsequent weight gain.	The dietary challenges were acute, follow-up weight data were available for only part of the cohort, and the observational associations with subsequent weight change do not establish causality.
McDougal et al., 2020 [31]	8 healthy adults	Isocaloric high-fat versus low-fat dinner	Two randomized 12-h overnight chamber stays in a crossover design	EE, RER dynamics, substrate oxidation, RER:FQ ratio, fat balance	The two meals produced distinct overnight RER and substrate-oxidation profiles without a significant difference in EE. The high-fat dinner resulted in a positive 12-h fat balance, whereas fat balance after the low-fat dinner was close to zero.	The study included only eight participants, used an overnight rather than a full 24-h protocol, and was designed as a proof-of-concept investigation.
Heinitz et al., 2020 [41]	11 otherwise healthy adults with overweight or obesity	Six weeks of inpatient 50% caloric restriction	Repeated 24-h assessment at baseline, after 1 and 3 weeks of caloric restriction, and after completion of the 6-week restriction period followed by 1 week of weight stabilization	24-h EE, sleeping EE, adaptive thermogenesis, cumulative energy deficit, body-weight and fat-mass loss	Adaptive thermogenesis was detectable after 1 week of caloric restriction and persisted after the intervention. A greater early reduction in 24-h EE beyond that predicted by changes in body composition predicted a smaller cumulative energy deficit and less weight loss over 6 weeks.	Exploratory secondary analysis with a very small sample and no parallel control group.

Study	Population	Intervention or metabolic challenge	WRIC protocol	Main outcomes	Principal findings	Major limitation
Carnero et al., 2021 [35]	9 active normal-weight adults, 9 adults with obesity without T2D, and 23 adults with obesity and T2D	Standardized meals in all groups, 10-week supervised aerobic training in the T2D group	A 24-h chamber assessment at baseline in all groups, repeated after aerobic training in the T2D group	Sleeping RQ, postprandial RQ kinetics after a standardized high-carbohydrate breakfast, substrate oxidation, and indices of metabolic flexibility	Sleeping and postprandial RQ responses differed across metabolic phenotypes. Aerobic training increased the postprandial RQ slope in participants with T2D, indicating improvement in selected features of metabolic flexibility.	Small groups and no non-exercise control group for the training intervention; the longitudinal analysis was restricted to participants with T2D.
Unlu et al., 2024 [50]	44 healthy, weight-stable adults	Energy-balance diet and 24-h fasting, each followed by 24-h ad libitum vending-machine food access	Two 24-h chamber assessments: energy balance and fasting	24-h EE, RER, carbohydrate and fat oxidation, and subsequent ad libitum energy intake	Smaller fasting-induced reductions in RER and carbohydrate oxidation were associated with greater subsequent ad libitum energy intake, whereas the change in fat oxidation was not significantly associated with intake.	The metabolic challenge was short, subsequent energy intake was assessed in a controlled vending-machine setting, and the observed associations do not establish causality.
Perry et al., 2024 [51]	97 generally healthy adults; 94 had valid energy-balance chamber data	Energy balance, 24-h fasting, and five 200% overfeeding diets differing in fat, carbohydrate, and protein content	Serial randomized 24-h chamber experiments during an approximately 40-day inpatient protocol, with washout periods between dietary conditions	24-h EE, RQ, fat, carbohydrate and protein oxidation, and circulating metabolomic profiles	High-carbohydrate overfeeding produced the greatest increase in 24-h EE. Changes in substrate oxidation reflected macronutrient availability and were coordinated with biologically consistent changes in circulating metabolites.	Not every participant completed every dietary condition, the population was predominantly male and metabolically healthy, and metabolomic data were integrated secondarily across a complex inpatient protocol.

McDougal et al., 2025 [32]	8 adults without major metabolic disease	Repeated high-fat dinner challenge and a two-step hyperinsulinemic–euglycemic clamp	Two 12-h overnight chamber stays separated by 5–7 days	Overnight RER kinetics, substrate oxidation, RER: FQ-based indices, and test–retest reliability	Several RER-based indices showed acceptable test–retest reliability. Meal-derived indices were not significantly associated with clamp-derived metabolic flexibility, indicating that the two challenges may capture different physiological dimensions.	The sample was very small and demographically homogeneous, the chamber protocol was limited to the overnight period, and no prospective clinical outcomes were assessed.
**Study**	**Population**	**Intervention or metabolic challenge**	**WRIC protocol**	**Main outcomes**	**Principal findings**	**Major limitation**
Ravussin et al., 2025 [56]	55 adults with obesity without T2D	Tirzepatide, titrated to 15 mg once weekly, or placebo during an 18-week calorie-restriction intervention	Randomized, double-blind trial with 24-h chamber assessments at baseline and week 18	Sleeping metabolic rate, 24-h chamber EE, sleeping and 24-h RER, substrate oxidation, body composition, appetite, and energy intake	Tirzepatide did not significantly attenuate metabolic adaptation after adjustment for changes in body composition. It increased fat oxidation and reduced appetite and energy intake compared with placebo.	This was a phase 1 mechanistic study with a modest sample size and an 18-week follow-up; differences in achieved weight loss complicated comparisons of body-composition-adjusted outcomes.
Basolo et al., 2025 [55]	11 adults with obesity, including 8 women	Six months of liraglutide treatment at clinically titrated doses	Prospective study with repeated 24-h chamber assessments before treatment and after 6 months	24-h chamber EE, sleeping metabolic rate, carbohydrate, fat, and protein oxidation, body weight, and body composition	Liraglutide treatment produced substantial weight and fat-mass loss without a significant change in 24-h chamber EE or sleeping metabolic rate. Fat oxidation increased, carbohydrate oxidation decreased, and protein oxidation remained unchanged.	This was a small, uncontrolled study with clinically titrated dosing; therefore, the observed metabolic changes cannot be separated from the effects of weight loss or attributed specifically to liraglutide.
Tam et al., 2016 [58]	30 adults with severe obesity: RYGB n = 5, SG n = 9, LAGB n = 7, low-calorie diet n = 9	Self-selected RYGB, SG, or LAGB, or an 800-kcal/day low-calorie diet for 8 weeks followed	23-h whole-room calorimetry, with EE extrapolated to 24 h, at baseline, 8 weeks, and 1 year	24-h EE, sleeping EE, resting metabolic rate, metabolic adaptation adjusted for	Body-composition-adjusted 24-h EE decreased in all groups at 8 weeks, but this reduction persisted at 1 year only after RYGB and SG. The magnitude of metabolic adaptation did not differ significantly between treatment groups.	Prospective observational, non-randomized design with self-selected treatment; small and unequal groups and substantial differences in achieved weight loss.

		by longer-term dietary management.	after treatment initiation	changes in body composition, body weight and composition, and cardiometabolic markers		

Study	Population	Intervention or metabolic challenge	WRIC protocol	Main outcomes	Principal findings	Major limitation
Basolo et al., 2018 [53]	80 adults with obesity without diabetes; 24 had complete week-24 chamber and body-composition data	Subcutaneous exenatide 10 μg twice daily or placebo for 24 weeks	Randomized, double-blind, placebo-controlled trial with 24-h chamber assessments at baseline, after treatment initiation, and at week 24 in a subset of participants	24-h chamber EE, sleeping metabolic rate, awake-fed thermogenesis, spontaneous physical activity, RQ, substrate oxidation, ad libitum energy intake, and body weight	Exenatide markedly reduced ad libitum energy intake shortly after treatment initiation but did not significantly affect 24-h chamber EE or substrate oxidation. The effect on energy intake diminished over time, and body-composition-adjusted 24-h chamber EE did not differ from placebo at week 24.	Only 24 participants had complete week-24 chamber and body-composition data, substantially limiting the precision of the long-term comparisons.
Corbin et al., 2023 [54]	35 adults with overweight or obesity: GLP-1/glucagon receptor agonist SAR425899, n = 17; placebo, n = 18	A 1000-kcal/d energy-deficit diet combined with escalating doses of the GLP-1/glucagon receptor agonist SAR425899 (0.06–0.20 mg/d) or placebo for 19 days	Three consecutive 24-h chamber assessments at baseline and three consecutive assessments on treatment days 17–19	Sleeping metabolic rate, 24-h chamber EE, RER, carbohydrate and fat oxidation, ketogenesis, body weight, and body composition	SAR425899 attenuated the reduction in body-composition-adjusted sleeping metabolic rate but did not alter metabolic adaptation in 24-h chamber EE. Fat oxidation and ketogenesis increased more with SAR425899 than with placebo.	The intervention lasted only 19 days, dropout was greater in the SAR425899 group, and the absence of a GLP-1 receptor agonist-only comparator prevented separation of the glucagon-receptor contribution.

RQ is retained where used in the original publications to describe whole-body respiratory gas exchange; the corresponding measure is termed RER in the main text. RQ, respiratory quotient; RER, respiratory exchange ratio.
